# Functional Roles of Protein Nitration in Acute and Chronic Liver Diseases

**DOI:** 10.1155/2014/149627

**Published:** 2014-04-30

**Authors:** Mohamed A. Abdelmegeed, Byoung-Joon Song

**Affiliations:** Section of Molecular Pharmacology and Toxicology, Laboratory of Membrane Biochemistry and Biophysics, National Institute on Alcohol Abuse and Alcoholism, 9000 Rockville Pike, Bethesda, MD 20892-9410, USA

## Abstract

Nitric oxide, when combined with superoxide, produces peroxynitrite, which is known to be an important mediator for a number of diseases including various liver diseases. Peroxynitrite can modify tyrosine residue(s) of many proteins resulting in protein nitration, which may alter structure and function of each target protein. Various proteomics and immunological methods including mass spectrometry combined with both high pressure liquid chromatography and 2D PAGE have been employed to identify and characterize nitrated proteins from pathological tissue samples to determine their roles. However, these methods contain a few technical problems such as low efficiencies with the detection of a limited number of nitrated proteins and labor intensiveness. Therefore, a systematic approach to efficiently identify nitrated proteins and characterize their functional roles is likely to shed new insights into understanding of the mechanisms of hepatic disease pathophysiology and subsequent development of new therapeutics. The aims of this review are to briefly describe the mechanisms of hepatic diseases. In addition, we specifically describe a systematic approach to efficiently identify nitrated proteins to study their causal roles or functional consequences in promoting acute and chronic liver diseases including alcoholic and nonalcoholic fatty liver diseases. We finally discuss translational research applications by analyzing nitrated proteins in evaluating the efficacies of potentially beneficial agents to prevent or treat various diseases in the liver and other tissues.

## 1. Introduction

Nitric oxide (NO) is a common free radical that is synthesized via enzymatic and nonenzymatic reactions in various cells and tissues. NO is also a very important intracellular signaling molecule in all vertebrates and even in plants. NO is enzymatically synthesized through three different isoforms of NOS (nitric oxide synthase), namely, NOS1, NOS2, and NOS3 [1]. Neuronal NOS (nNOS or NOS1) is expressed in the brain in large quantities [2, 3]. NOS2, known as inducible NOS (iNOS), is induced in various tissues in response to proinflammatory cytokines or oxidative stress under pathological conditions or following exposure to toxic agents [4]. NOS3, known as endothelial NO synthase (eNOS), is typically expressed in vascular endothelial cells and associated to plasma membrane [4]. Thus the three NOS isozymes were named either after their constitutive expression in certain tissues (nNOS and eNOS) or after their expression mechanism (iNOS). While both NOS1 and NOS3 can be activated by intercellular calcium and calmodulin, NOS2 can be induced completely with normal levels of calcium [4]. NO is produced by a reaction that is composed of two sequential steps: (1) the hydroxylation of guanidino nitrogen of L-arginine, leading to the generation of the intermediate N^*ω*^-hydroxy-L-arginine (NOHA), and (2) NOHA is then oxidized to NO and L-citrulline [5]. NO by itself is not highly reactive since NO is the intermediate molecule between molecular oxygen (O_2_) and nitrogen (N_2_) [4]. NO has a very short half-life; however, it can diffuse freely across cell membranes [6]. The classical NO signaling is related to its activation of soluble guanylate cyclase (sGC) and subsequently cyclic guanosine monophosphate- (cGMP-) dependent protein kinases [7]. The less- and nonclassical NO signaling pathways are related to NO binding to cytochrome c oxidase in the mitochondria and the cGMP-independent NO-related posttranslational modifications (PTMs), respectively [7].

**Figure 1:**
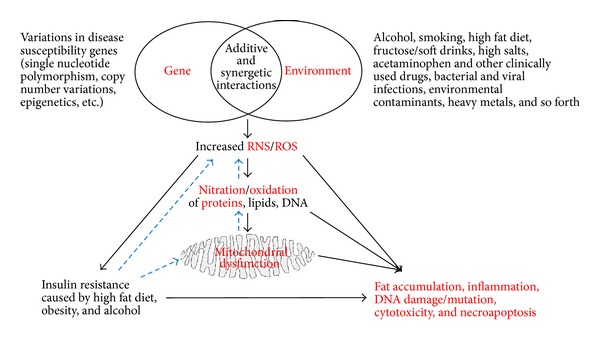
Synergistic interaction between genetic and environmental factors in promoting acute and chronic liver diseases. Additive or synergistic interactions between genetic and environmental factors such as alcohol, smoking, fat diet, and other potentially toxic substances result in increased production of RNS/ROS, which can modify cellular DNA, lipids, and proteins, promoting mitochondria dysfunction and interrupting many important signaling pathways. Continued presence of increased nitroxidative stress (through a vicious cycle shown in blue dotted arrows) contributes to acute and chronic liver diseases including alcoholic fatty liver disease (AFLD) and nonalcoholic fatty liver disease (NAFLD).

There are many forms of NO which are interchangeable and leading to the production of reactive nitrogen species (RNS) such as nitrosonium cation (NO^+^), nitroxyl radical (NO^·^), nitroxyl anion (NO^−^), nitrite (NO_2_^−^), nitrate (NO_3_^−^), and nitrous oxide (N_2_O). These intermediates provide the NO molecule as a unique signaling molecule that can act within the cell or interact with adjacent cells without the need of a receptor [6]. The generation of secondary oxides of nitrogen such as peroxynitrite (ONOO^−^) and nitrosothiols (RSNO) can also produce diverse biological effects through interactions with cellular macromolecules (e.g., DNA, lipids, and proteins), as illustrated in Figure 1. Peroxynitrite can stimulate nitration of tyrosine (Tyr) residue(s) and* S*-nitrosylation of Cys residues, both of which can lead to alterations of protein structure and function [8, and references therein]. Nitrogen dioxide (NO_2_) can also interact with other oxidants such as superoxide radicals (O_2_^·^−^^), H_2_O_2_, and transition metal centers in various heme-containing proteins, leading to production of peroxynitrite, which nitrates Tyr residues of various proteins generating 3-nitroTyr (3-NT), which is widely accepted as a foot print of peroxynitrite formation [9–11]. Reactive oxygen species (ROS) including superoxide radicals can be produced from various sources including mitochondrial electron transport chain (ETC) and other cellular enzymes such as NADPH oxidase and myeloperoxidase or eosinophil oxidase in immune cells (i.e., macrophage cells and neutrophils), ethanol-inducible cytochrome P450 2E1 (CYP2E1) and CYP4A isozymes in endoplasmic reticulum (ER), cytosolic xanthine oxidase, and so forth [12–19]. RNS not only interact with Tyr, but also with tryptophan (Trp) [20], lipids [21], and vitamins [22, 23]. Thus, the nitrative modifications of target proteins, DNA, lipids, and vitamins, usually contributing to alterations of their normal functions and the development or progression of tissue injury [4, 24, 25]. Tyr is a common amino acid found in most proteins [26] and is readily an accessible target for protein nitration since it is often exposed on the surface of the protein due to its mild hydrophilic characteristic [27, 28]. Thus, protein nitration data in literature is largely associated with Tyr nitration and functional consequences. The modification of Trp residues in proteins may occur in a more limited number of sites than that of Tyr residues since Trp is less abundant than Tyr in the protein and is usually buried inside with an exception for a few surface exposed Trp residues. Therefore the modification of those Trp residues may alter specific interaction of proteins and/or enzymes with other molecules, which may cause functional dysregulations [20]. However, in this review, we do not discuss much about Trp nitration since its formation, identification, and significance were extensively reviewed elsewhere [20, 29, 30]. It is likely that modification of each amino acid (e.g., nitration of Tyr or Trp or* S*-nitrosylation of Cys) seems to depend on the local microenvironment such as pH, solvent exposure or accessibility, peptide loop structure, and the presence of other competing amino acids (e.g., Cys near the potentially nitrated Trp or Tyr) [20] or a denitrase present in several tissues [31].

Protein Tyr nitration has been reported in correlation with many pathological conditions such as cardiovascular disorder, diabetes, hepatic disease, ischemia-reperfusion (I-R) injury, neurodegenerative diseases, stroke, inflammatory diseases, and cancer, as reported in many human diseases and experimental models including cell culture systems [4, 32]. Despite the numerous reports about the occurrence and consequence of Tyr nitration in various human diseases and in experimental animal models [4, and references therein], there have been a relatively small number of reports that systematically dealt with identification and functional characterization of nitrated proteins in various subcellular organelles, including mitochondria, especially under conditions with increased nitroxidative stress. Fewer reports on protein oxidation, nitrosation, and nitration might be due to the requirement for specific reagents, the lack of suitable methods to systematically identify and purify nitrated proteins, and the relatively late development and advancement of highly sensitive mass spectrometry (MS) instruments. There are many questions that need to be addressed when working with nitrated proteins as follows: (1) what are the sources of ROS/RNS in enhancing protein nitration? (2) which proteins are nitratively modified? (3) are their activities/functions altered following nitrative modifications? (4) what are the overall implications of nitrated proteins in mitochondrial dysfunction or other organelles in various disease states including hepatic liver disease/injury? (5) can the nitrative protein modifications and subsequent dysfunction be prevented with a potential therapeutic agent? and (6) most importantly, do we have a suitable method to efficiently identify the nitrated proteins to study the causal relationship between protein nitration and mitochondrial dysfunction and tissue injury? In this review, we discuss the roles of nitrative stress and protein Tyr nitration in the development and/or progression of various stages of liver disease. We specifically focus on nitration of mitochondrial proteins and their functional implications. We also briefly discuss various methods for identifying nitrated proteins in experimental models with emphasis on the immunoaffinity purification of nitrated proteins followed by their identification by MS analysis. Finally we briefly discuss the utility of studying nitrated proteins in different subcellular fractions in various tissues as well as future translational research opportunities.

## 2. Functional Consequences of Protein Nitration in Acute and Chronic Liver Diseases

Many pathological conditions usually result from changes in genetic or environmental factors or a synergism between both factors. Increased nitroxidative stress seems to play a critical role in mediating the pathological effects of this synergism or even each factor alone (Figure 1). Under normal physiological conditions, protein nitration and function alterations could be properly managed by the antioxidant host defense system [50, 51] increased nitroxidative stress, four possible outcomes may arise from protein nitration: (A) a decrease or loss, (B) an increase, (C) immunomodulation and immunogenicity, or (D) little or no effect on the biological functions of the modified target proteins. One of the challenges that arise when determining the functional implications of protein nitration is that nitration is not unique to Tyr residues since Trp can be also nitrated. In addition, various amino acids such as Trp, cysteine (Cys), histidine (His), proline (Pro), lysine (Lys), and methionine (Met) can be also oxidized or* S*-nitrosylated [20, 50]. Another challenge could be to identify and characterize nitrated proteins that are expressed in very low quantities such as various transcription factors and signaling proteins, contributing to tissue injury.

### 2.1. Inhibition of Nitrated Proteins and Functional Consequences

The decreased function by protein nitration may result from the inhibition or loss of catalytic activity and/or decreased protein levels since protein nitration likely changes the protein secondary structure, hindering the access of the substrate to the activity site [4]. Alternatively, nitrated and/or oxidatively modified proteins that are improperly folded or damaged are known to be removed through ubiquitin-dependent proteolytic degradation mechanism [52, 53] while trypsin, serine proteases, or calpain is not involved in this process [53]. In addition, nitration and/or oxidation may lead to protein aggregation and the consequent loss of its activation and/or functions reported with *α*-synuclein, a major constituent of the typical protein aggregates observed in several neurodegenerative diseases that are collectively referred to as synucleinopathies [54, 55]. The removal of nitrated proteins can serve as a defense mechanism against nitroxidative stress-related harmful consequences but also plays an essential role in many vital processes including cell division, apoptosis, cell differentiation, DNA repair, membrane transport, oncogenesis, and signal transduction [56, 57]. At the same time, degradation with decreased levels of essential proteins for cell maintenance and survival can also be detrimental to the cells, especially when the degradation rates of proteins essential for energy production, antioxidant or anti-inflammatory defense, urea metabolism, and so forth, exceed those of their regeneration or other compensatory mechanism(s) due to persistence of toxic effects.

By using biotin-*N*-maleimide (biotin-NM) as a sensitive biotin-switch probe to proficiently identify oxidized proteins, we previously showed that many mitochondrial proteins including the enzymes involved in fat oxidation and energy supply could be oxidatively modified under increased nitroxidative stress and thus inactivated, leading to increased fat accumulation and ATP depletion in the experimental models of alcoholic fatty liver disease (AFLD) and acute I-R liver injury (Figure 1). We expect that protein nitration would also produce similar or more damaging effects in combination with other PTMs. We briefly describe the inhibition of catalytic activities and/or degradations of essential proteins following nitration and their functional implications in three major liver disease/injury models, namely, (1) AFLD, (2) nonalcoholic fatty liver disease (NAFLD), and (3) drug/xenobiotic-induced acute liver injury.

#### 2.1.1. Alcoholic Fatty Liver Disease

The AFLD ranges from simple steatosis with microvesicular fat accumulation to more severe forms including steatohepatitis, fibrosis, cirrhosis, and finally hepatocellular carcinoma following chronic heavy alcohol ingestion [16, 58–62]. Obesity, hyperlipidemia, inflammation, nitroxidative stress, insulin resistance, and so forth are risk factors for AFLD ([63, 64] and references therein). Several mouse models have been used to evaluate the effect of protein nitration on nitroxidative stress-mediated in AFLD. For instance, the role of protein nitration has been studied in mouse strains with ablated genes that are involved in either decreasing or increasing the levels of superoxide and NO. The mouse strains include* Cyp2e1*(−/−), SOD1(−/−),* SOD2*(−/−),* iNOS*(−/−), and TNF-*α* receptor (*TNFR*) (−/−), as described below.

Peroxynitrite and protein nitration were suggested to be main causes of acute and chronic alcoholic fatty liver injury models. Expression of iNOS was increased following the exposure to ethanol [15, 16, 37, 65–67]. Administration of the Lieber-DeCarli ethanol liquid diet has significantly increased the levels of steatosis, apoptosis, necrosis, and inflammation in wild-type (WT) mice compared to the corresponding* iNOS*(−/−) mice. The severity of liver injury was proportional to the levels of hepatic nitration and inhibition of mitochondrial function in alcohol-exposed WT mice, while* iNOS*(−/−) mice with markedly decreased levels of nitrated proteins were resistant to AFLD [15, 16] and that protein nitration was shown to inhibit complex I (NADH ubiquinone oxidoreductase) and complex V (ATP synthase) activities in acute and chronic alcohol-exposure models [36, 37, 68]. Thus protein nitration seems to play an important role in promoting the ethanol-mediated mitochondrial dysfunction and liver injury since these proteins are essential for normal mitochondrial function while protein nitration might lead to irreversible modification of the respiratory chain proteins, contributing to increasing mitochondrial sensitivity to NO and ultimately AFLD [69]. We and other laboratories clearly showed that nitration of ATP synthase led to significant inhibition of its activity in ethanol fed rats and mice [37, 70]. The suppression of ATP production would certainly increase mitochondrial sensitivity to other oxidative insults and necrotic injury. In an acute binge ethanol model, iNOS expression, serum nitrite/nitrates, increased protein nitration of mitochondrial complex V (ATP synthase), decreased activities of both complex I and V, and mitochondrial DNA depletion were observed in WT mice but not in* SOD2*(−/−) mice [71]. The authors suggested that these damaging effects were probably due to protein nitration since administration of iNOS inhibitors and peroxynitrite scavengers like uric acid ameliorated the ethanol-induced nitration, inhibition of activity, and mitochondrial depletion of ATP synthase. In addition, mice lacking SOD2, which would scavenge superoxide and thus block the peroxynitrite formation, exhibited prolonged mitochondrial DNA depletion while mice overexpressing SOD2 showed opposite outcomes [36]. Similar to the protective role of mitochondrial SOD2, cytosolic SOD1 also exhibits protective roles against ethanol-mediated hepatic damage. It has also been shown that, in mice deficient in SOD1, the levels of protective hepatic ATP content and SOD2 expression were decreased while oxidative damage and nitro-Tyr formation were elevated in response to ethanol feeding, leading to greater hepatic injury in the* SOD1*(−/−) mice [61]. This data suggests that mitochondrial dysfunction in the liver following ethanol exposure might originate from the compromise in the cytosolic antioxidant defense mechanism. In alcohol consumption studies, it was suggested that ethanol increased the sensitivity of mouse and rat hepatocytes to the combined effects of ethanol and NO [36, 71–73]. This combined effect of ethanol and NO increases the susceptibility to hypoxia and depression of mitochondria bioenergetics reserve energy state of the liver, leading to energy depletion and ultimately the development of alcohol-mediated hepatic injury [72, 73]. While NO donors inhibited mitochondria respiration and increased mitochondrial dysfunction in ethanol-fed rats more than the controls [73],* iNOS*(−/−) exhibited less severe liver injury with decreased levels of hypoxia-inducible factor 1-*α* in the perivenular region of the liver lobule than in the corresponding WT [16, 72]. Taken together, hepatic mitochondria from ethanol-fed mice or rats are more sensitive to NO and RNS while iNOS plays an essential role in determining the response to hypoxic stress in vivo. Since ethanol hepatotoxicity was also significantly prevented through a mechanism that involves a decreased inflammatory response, tumor necrosis factor-*α* (TNF-*α*) formation, and fatty liver [15], it was not surprising that* TNFR*(−/−) mice exhibited significantly less severe ethanol-mediated hepatotoxicity accompanied with markedly lower levels of protein Tyr nitration than those of their WT counterparts.

In addition, the elevated nitroxidative stress and subsequent hepatotoxicity in acute and chronic alcohol exposure models are correlated with the increased hepatic amounts and catalytic activities of CYP2E1, the most relevant cytochrome P450 for the development and progression of AFLD [13, 74–80]. For instance, in the chronic alcohol-fed mouse models, the levels of protein nitration were highest in* Cyp2e1* knock-in mice, followed by WT mice and lastly in* Cyp2e1*(−/−) mice, which exhibited almost basal levels of nitrated proteins [81]. The levels of protein nitration correlated well with the increased levels of hepatic transaminases, steatosis, and necrosis [81]. Since CYP2E1 is highly expressed in the endoplasmic reticulum (ER) and mitochondria [82–85], where iNOS is also present, it is conceivable to predict that protein nitration and oxidation can take place in both organelles and their participation in the development of AFLD either through increased ER stress and/or mitochondrial dysfunction, both of which are known as causative factors of AFLD.

Formation of peroxynitrite and protein nitration might not be the only causes for ethanol-induced hepatic toxicity. For instance, induction of nitrative stress along other forms of oxidation in intestinal epithelium may also serve as a cause for ethanol-induced gut leakiness and endotoxemia, leading to inflammatory liver injury. The intestinal epithelium, under normal conditions, serves as a highly selective barrier so that potentially toxic substances or products (e.g., lipopolysaccharide (LPS)) of the gut bacteria into the circulation [86–88]. Disruption of the intestinal barrier integrity (i.e., leaky gut) may lead to the penetration of luminal bacterial products such as endotoxin into the mucosa, then into the systemic circulation, and initiate local inflammatory processes in the intestine, blood vessels, and even in the liver [88]. Numerous studies in the literature showed that ethanol can compromise the integrity of intestinal epithelium, leading to increased gut leakiness in rodents [89–95] and human alcoholics [90, 96]. Ethanol-induced production of NO via induction of iNOS and subsequent formation of peroxynitrite in intestinal Caco-2 cells lead to barrier dysfunction probably due to oxidation and nitration of cytoskeletal proteins and/or tight junction proteins [88, 90]. This data was also validated in vivo studies in a mouse model fed ethanol for 10 weeks with iNOS inhibitors. Coadministration with iNOS inhibitors attenuated ethanol-mediated NO overproduction, oxidative tissue damage, leaky gut, endotoxemia, and liver injury including steatosis [97].

CYP2E1 protein expression was also increased in Caco-2 intestinal epithelial cells and in both acute and chronic ethanol-exposed mouse models [78, 98]. In addition to increased nitroxidative stress and protein nitration, expression of redox-sensitive circadian clock proteins CLOCK and PER2 was significantly elevated in Caco-2 cells and in mice exposed to ethanol, leading to increasing intestinal hyperpermeability [98]. The usage of a CYP2E1 siRNA in Caco-2 cells or antioxidant* N*-acetyl cysteine (NAC) in mice exposed to ethanol ameliorated nitroxidative stress including iNOS expression and consequently inhibited the expression of CLOCK and PER2 as well as gut permeability. Further, recent studies from our laboratory showed that iNOS and protein nitration were also increased in intestinal epithelial cells in binge ethanol-exposed WT but not in the corresponding* Cyp2e1*(−/−) mice, leading to increased endotoxemia and subsequently inflammatory liver damage and apoptosis in ethanol-exposed WT mice [78]. We then showed that inhibition of iNOS induction and protein nitration of intestinal epithelial cells in mice pretreated with a CYP2E1 inhibitor or an antioxidant NAC ameliorated all the damaging effects in intestinal epithelial cells accompanied by decreased serum endotoxin levels and oxidative hepatic injury including steatosis and apoptosis in the ethanol-exposed WT mice. In contrast, these ethanol-mediated events were markedly attenuated in alcohol-exposed* Cyp2e1*(−/−). These results support at least a partial role of intestinal protein nitration in mediating the alcohol-induced gut leakiness and subsequent hepatic injury. Protein nitration in intestine cells is also likely to affect the cytoskeleton protein architecture [90] and/or intestinal tight gap-junction proteins, leading to alteration of barrier function with increased permeability [78, 97]. Collectively, all these studies suggest that elevated iNOS and CYP2E1 play an important role, at least partially, in producing peroxynitrite and protein nitration in gut leakiness and AFLD, while normal levels of SOD1 and SOD2 seem protective against the development of AFLD since ethanol-mediated hepatic toxicity was worsened upon the inhibition or deletion of these SOD proteins. In addition, the development of peroxynitrite and subsequent protein nitration of essential mitochondrial proteins and other vital proteins in other subcellular organelles (e.g., cytosol) would be expected to play a causal or contributing role in mediating ethanol-mediated hepatotoxicities (Table 1). This data also suggests that in liver exposed to toxic substances/xenobiotics that increase ROS/RNS would sensitize the liver for even stronger and faster development of ethanol-mediated damaging effects, as evidenced with concurrent exposure to LPS, acetaminophen (APAP, a major ingredient of Tylenol), nicotine (a major component of smoking), 3,4-methylendioxymethamphetamine (MDMA) ([38, 99] and references within), high fat diet (HFD), and so forth. However, other forms of PTM including lipid peroxidation,* S*-nitrosylation including glutathionylation, glycosylation, and glycation including advanced glycation end-products (AGE), might also be involved in AFLD [8, 100] (Figure 1).

#### 2.1.2. Nonalcoholic Fatty Liver Disease

NAFLD, usually caused by chronic ingestion of nonalcoholic substances such as HFD, cholesterol-containing fast food western diet, fructose, and choline-deficient diet, is one of the most common chronic liver diseases in the USA and developed countries [101, 102]. NAFLD often associated with overeating and obesity starts as simple steatosis and can progress to inflammation (nonalcoholic steatohepatitis, NASH), fibrosis, cirrhosis, and even cancer, as similar to the progression of AFLD [13, 103]. Many risk factors are known to promote the progression of NAFLD to NASH, fibrosis, and hepatocarcinoma. The risk factors include increased nitroxidative stress, mitochondria dysfunction, insulin resistance, disturbance of fat homeostasis, gut leakage, cytokine, and immune dysregulation [103, 104]. There are numerous reports in the literature showing that increased levels of iNOS, CYP2E1, and protein nitration play an important role, at least partially, in the development and/or progression of NAFLD, as shown in various experimental models and people with NASH ([74, 105–115], and references therein). In a NASH model fed a HFD for 20 weeks, the overexpression of tissue inhibitor of metalloproteinase (TIMP3) in macrophages ameliorated HFD-induced insulin resistance, adipose inflammation, and NAFLD probably through the inhibition of various parameters of nitroxidative stress including hepatic protein nitration [116]. The role of leptin in mediating the progression of NAFLD to NASH was evaluated in WT and leptin-deficient* ob/ob* mice fed a HFD for 16 weeks followed by treatment with carbon tetrachloride (CCl4) [117]. HFD-mediated NASH observed in WT was resulted from the induction of iNOS and NADPH oxidase, leading to increased nitroxidative stress, which then activated hepatic Kupffer cells with increased inflammation [117]. In addition, the coadministration of leptin with CCl4 to* ob/ob* mice significantly elevated nitroxidative stress compared to treatment with CCl4 alone [117]. Finally, the deletion of iNOS or p47 phox subunit of NADPH oxidase ameliorated the peroxynitrite formation and protein nitration [117], suggesting an important role of protein nitration, at least partially, in the leptin-mediated activation of Kupffer cells. Further, metal iron was also found to increase liver injury in diabetic rats, partially, through nitration of glucokinase accompanied with decreased levels of its expression and activity, shedding some new lights on the role of iron in the development of hepatotoxicity in diabetes mellitus [118]. Peroxynitrite and protein nitration were also reported to play a central role in the suppression of the mitochondrial respiratory chain activity, leading to mitochondrial dysfunction in a NASH model using* ob/ob* mice while these effects were ameliorated by melatonin administration [119]. The source of NO seems to be important in determining the outcome of the increased or decreased NO levels. For instance, in a HFD-mediated NAFLD rat model, simvastatin was found to protect against the development of HFD-induced liver fibrosis via differentially regulating NOS isozymes, where eNOS was found to be elevated while iNOS was decreased [112]. These results suggest that eNOS seems to be protective against NAFLD, while iNOS seems to promote NAFLD [112]. Protein nitration was also reported to play an important role in the development of insulin resistance in NAFLD in WT mice infused for 6 h with a 20% intralipid emulsion [120]. Further, iNOS induction and consequent Tyr-nitration of key insulin signaling proteins such as AKT, insulin receptor-*β* (IR*β*), insulin receptor substrate-1 (IRS-1), and insulin receptor substrate-2 (IRS-2) [120] lead to the interference with Tyr phosphorylation, a signature feature of the insulin signaling pathways. In addition, there was increased serine phosphorylation, which leads to the inhibition of the hepatic insulin signaling pathway, especially when Tyr phosphorylation was inhibited [120]. These events were monitored with minimal changes in the basal levels of the insulin signaling molecules before and after lipid infusion [120]. Thus, a novel mechanism for hepatic insulin resistance caused by circulating lipids from HFD through protein nitration was suggested since these events were significantly ameliorated in* iNOS*(−/−) mice [120]. Furthermore, nitration of IRS-1 and IRS-2 proteins caused decreased expression of both proteins in obese diabetic rats or when hepa1c1c7 and HepG2 hepatoma cells were exposed to NO donors such as GSNO and SIN-1 in a time- and dose-dependent manner. In contrast, their expressed levels were increased after the hepatoma cells or obese diabetic rats were treated with an iNOS inhibitor, N6-(1-iminoethyl)-L-lysine (L-NIL) compared to their control [44]. The decreased protein levels of IRS-1 and IRS-2 following their nitration in these models of NAFLD might be due to their ubiquitin-dependent degradation, as discussed previously [53, 120]. Taken together, the outcome of these effects would depend not only on the extent of inactivation and/or loss of the nitrated proteins but also on the cellular ability to overcome the loss of these particular nitrated proteins by various cell defense mechanisms. Thus, it is reasonable to conclude from the two aforementioned studies [44, 120] on HFD-mediated hepatic insulin resistance where insulin signaling molecules could be by nitrated without change in their protein levels probably occurring at early stages of the disease process (i.e., following a short-term exposure). Alternatively, insulin resistance could also result from the decreased levels of nitrated insulin signaling molecules via proteolytic degradation, usually observed at later time points following exposure to persistent insults.

Based on the aforementioned reports, future studies on the functional implications of protein nitration should consider the temporal evaluation of protein nitration and the remaining levels of nitrated proteins, since the nitration process is extremely dynamic and that the levels of nitrated proteins could vary dramatically over the course of acute or chronic hepatic insults. This is particularly important since there are many reports suggesting that protein nitration is not chemically stable, as previously believed. By using a reducing agent sodium dithionite as described previously [121], we showed that most of the nitrated cytosolic proteins, determined by immunoblot analysis following 2D gel electrophoresis, completely disappeared in APAP-exposed mouse livers [40]. The disappearance of nitrated protein spots was due to the reduction of nitro-Tyr to amino-Tyr, which was no longer recognized by the antibody to 3-NT. The reduction of nitro-Tyr to amino-Tyr was also reported by a pure chemical reaction from Fe3+-containing heme as in hemoglobin and myoglobin in the presence of a reducing agent [122]. Similar reaction has been also reported under physiological conditions where nitrated deoxynucleo bases were reduced to their amino analogues [123].

One might think that the decreased levels of nitrated proteins over time only result from their proteolytic degradation, as previously discussed; however, this is not necessarily true. For instance, in the presence of protease inhibitors to block proteasomal degradation of nitrated proteins, nitrated BSA disappeared when incubated with the homogenates of spleen or lung tissues, but not with the homogenates of rat liver or kidney, suggesting that different isoforms of denitrase might exist in a tissue-specific manner [124]. Protein with denitrase activity has been detected in a variety of tissues including the liver, heart, lung, brain, spleen, and kidney [31, 125, 126]. Denitrase activity has been suggested to be present in both aqueous and membrane phases in the cells [31]. Indeed, denitration reaction via denitrase was observed with nitrated Histone H1.2 [125], glutamine synthetase [127], calmodulin [128], L-type Ca^2+^ channel [129], cyclooxygenase [31], and so forth. Although constitutively active denitrase was present in many tissues and partially purified using the nitrated cyclooxygenase as a substrate [31, and references therein], the biochemical and regulatory properties of denitrase enzyme still need additional investigations. Collectively, data from these reports suggested that denitration may serve as an adaptive mechanism by which cells can repair damaged proteins. In addition, the nitration and denitration process can be of particular importance for cell signaling-related events. For a molecule to act as a signal, nitration-denitration has to be a reversible process. Indeed, nitrated cytochrome c was suggested to act as a signal molecule, as shown in cytochrome c overexpressing HeLa cells exposed to peroxynitrite with the spontaneous translocation of nitrated cytochrome c from mitochondria to cytosol and nucleus [130]. It is also very important to temporally study the nitric oxide bioavailability through the course of NAFLD. For example, in a mouse model of NAFLD fed a HFD for 8 and 16 weeks, NO contents were initially increased causing mitochondrial damage with mitochondrial protein alterations but decreased at later stages of the NAFLD [131]. The authors suggested that decreased NO might be involved in the progression of NAFLD to NASH. Again the decreased NO levels at later stages seem to be due to increased levels of arginase-1, depleting the substrate (i.e., L-arginine) of NOS and decreased levels of activated eNOS-PSer1177, which is protective against disease progression [112, 131]. However, in a clinical study using patients with different grades of liver cirrhosis, a positive correlation between the levels of nitrated proteins in plasma, platelets, and liver tissues and the severity of liver cirrhosis was found [132]. Thus, based on the results from previous reports [124, 132] and others, it is still unclear whether protein nitration can directly play a role in the initiation and/or development of NASH progressed from NAFLD. NAFLD is not only induced by pathological conditions such as obesity and diabetes but also by various diets such as HFD, fructose, and (methionine- and) choline-deficient diet [133–136]. Collectively these reports suggest a potential role of nitrated proteins in mediating NAFLD through various mechanisms (Figure 1).

#### 2.1.3. Acute Liver Injury Caused by Drugs and Xenobiotics

There are many enzymes that are expressed in the liver and other extrahepatic tissues while their activities are compromised by Tyr-nitration following exposure to certain drugs (e.g., APAP, zidovudine (AZT), MDMA, tamoxifen, amiodarone, and so forth) or other toxic substances including lipopolysaccharide (LPS). These agents can provoke acute liver injury with or without fat accumulation probably through increased nitroxidative stress-mediated inhibition of the enzymes involved in the fatty acid oxidation pathway (i.e., mitochondrial *β*-oxidation) [38, 137, 138]. If an enzyme, essential for the energy production or detoxification of certain metabolites, is inhibited under elevated nitroxidative stress, this inhibition is likely to exert deleterious effects on the liver function and/or hepatic disease progression. For instance, mitochondrial carbamoyl phosphate synthetase 1 (CPS1), which is responsible for the detoxification of excess ammonia, was found to be inactivated when it is nitrated at Tyr1450 [33]. This decline in CPS1 activity may result in hyperammonemia and subsequent mitochondrial dysfunction in in vivo situations, contributing to liver damage and possibly injury to the brain. LPS dramatically increased protein nitration along with other parameters of oxidative stress such as lipid peroxidation in the livers of peroxisome proliferator-activated receptor-*α* (PPAR*α*)(−/−) than in WT, which also exhibited significantly increased nitration compared to its untreated control, leading to elevated hepatic toxicity in* PPAR*α**(−/−) than WT [139]. Mitochondrial dysfunction was observed in LPS-exposed* PPAR*α**(−/−) mice, as evidenced by the inhibition of complex I and ATP synthase activity, both of which were reported to lose their activities by nitration [36, 139]. Further, LPS exposure decreased the catalytic activity and expressed amount of hepatic mitochondrial glutamine synthetase (GS) in rat liver through nitration at multiple Tyr residues including the active site Tyr160 [34], accompanied by inactivation. Since GS is also responsible for ammonia elimination, a compromise in its activity and function likely contributes to sepsis-induced development of hyperammonemia in patients suffering from hepatic cirrhosis [34]. The disturbance in ammonia metabolism may not only affect the liver but also the brain, leading to a condition known as the hepatic encephalopathy.

Mitochondrial ATP synthase is essential for providing cellular energy (i.e., ATP) for proper maintenance and survival of all living cells. If ATP synthase (mitochondrial complex V) is inhibited, this leads to depletion of an essential energy source, thus contributing to acute necrotic liver injury. We showed that the activity of ATP synthase was significantly inhibited in mice subjected to I-R injury [140], in MDMA-exposed rats [38], in LPS-exposed* Ppara*(−/−) mice [139], and in mice exposed to a toxic dose of APAP [35]. In these studies, we confirmed its nitration by immunoblot analysis with the anti-3-NT antibody following the immunoprecipitation of ATP synthase. It is noteworthy to mention that, in all of these models, the protein levels of ATP synthase seemed unchanged in response to any of those treatments, suggesting that the inhibition of its activity was mainly due to nitration-mediated inactivation.

Several hepatic enzymes involved in cellular defense were reported to be nitrated and their activities inhibited in livers and other tissues or cultured cells after exposure to toxic insults. APAP can be considered a prime example of the role of protein nitration in drug-induced liver injury (DILI). It has been shown that nitro-Tyr protein adducts formation correlated very well with the areas of hepatic necrosis [141]. The direct evidence of the critical role of peroxynitrite in APAP-mediating hepatic injury has also been provided by the seminal work of Knight et al. [142]. This study showed that the protective effects of GSH, either cotreated with APAP or administered at different intervals up to 2.25 h post-APAP treatment, were resulted from the restoration of cellular GSH levels and the increased efficiency of scavenging peroxynitrite [142]. Similar results were also obtained in glutathione-peroxidase deficient animals [142]. In addition, Cover et al. [143] showed for the first time that APAP can selectively promote mitochondrial protein nitration. This study also provided evidence that peroxynitrite can cause mitochondrial DNA damage directly and/or indirectly via the mitochondrial damage pathway [143]. The events for APAP-induced protein nitration and liver injury seem dependent on the presence of CYP2E1, which is one of the major enzymes involved in the metabolism of APAP [40, and references therein]. Furthermore, in a comprehensive study using a toxic dose of APAP, nitration of cytosolic Cu/Zn-dependent superoxide dismutase (SOD1) eventually led to ubiquitin-mediated degradation and significant inhibition of its activity and protein levels in WT but not in the corresponding* Cyp2e1*(−/−) mice, as evidenced by immunoprecipitation using the anti-SOD1 antibody followed by immunoblot analysis with either anti-3-NT or antiubiquitin antibody. These results suggest a role of CYP2E1 in protein nitration and subsequent degradation [40]. Further, several reports showed that when isolated cells or proteins were exposed to peroxynitrite, the induction of proteolytic degradation was observed [54, 55, 144, 145]. The proteolytic degradation of bovine SOD1 was also increased by the 20S/26S proteasome when Tyr108 was nitrated [53]. Taken together, protein nitration can contribute to APAP-mediated toxicity via nitration-mediated inactivation and/or proteasomal degradation of essential proteins in antioxidant defense. The inhibition of antioxidant mitochondrial SOD2 also plays a critical role in promoting APAP-induced hepatotoxicity due to its nitration and subsequent inactivation, hampering cell's ability to defend itself against increased nitroxidative stress [146]. In addition, nitration of cytosolic glutathione reductase (GR) at Tyr106 and Tyr114 decreases GR binding to its substrate oxidized glutathione (GSH) and thus markedly decreased its activity with increased amounts of oxidized GSH [42, 43]. Although this reaction was observed in an in vitro system, it is likely that, in case of increased RNS, hepatic GR may become one of the enzymes that can be also inhibited through nitration. Persistently high levels of oxidized GSH may lead to a vicious cycle of oxidative stress development and sensitize the target tissue to an additional insult. All these results suggest that protein nitration also plays an important role, at least partially, in liver injury caused by acute, subchronic, and chronic exposure to a variety of drugs and hepatotoxic substances. It should be noted however that removal of nitrated proteins either via ubiquitin-dependent protein degradation [40, 53] or autophagy-dependent clearance of damaged mitochondria [147] can also be considered a hepatoprotective pathway. Based on these studies, it is likely that the ultimate hepatic effects of a toxic substance could be decided by the delicate balance between the occurrence and removal of nitrated proteins.

### 2.2. Activation of Nitrated Proteins and Functional Consequences

Another consequence of protein nitration is a gain of normal protein functions that can either be beneficial or harmful. For example, nitration at Tyr33 or Tyr56 of the 90-kDa heat-shock protein (Hsp90) is likely to convert the Hsp90 into a toxic protein through a gain of function. Using an antibody that recognizes the nitrated Hsp90 [48], nitrated Hsp90 immunoreactivity was detected in motor neurons of patients suffering from amyotrophic lateral sclerosis (ALS), in an animal model of ALS, and in an experimental model of spinal cord injury [48]. The authors concluded that nitration of a single important protein Hsp90 can initiate cell death while this protein can also become a potential target for therapeutic intervention [48]. This observation is also of particular interest in understanding the role of CYP2E1 and the mechanism of acute and chronic liver injuries where elevated CYP2E1 is known to play an important role in mediating these injuries. Hsp90 was found to interact with membrane-bound CYP2E1 forming a binary complex, which then transfers the membrane-bound CYP2E1 to the proteasome complex for its degradation [148]. Ethanol was found to compromise this interaction between Hsp90 and CYP2E1, resulting in increased levels of CYP2E1 protein expression, which can damage the liver cells through its nitroxidative stress-mediated events [148]. These results are consistent with the inhibition of Hsp90 by geldanamycin, which increased CYP2E1-mediated toxicity in HepG2 hepatoma cells [149, 150]. Since both acute binge and chronic ethanol administration also promote hepatic protein nitration, it would be interesting to evaluate whether Hsp90 is nitrated in these alcohol-exposure models and whether this nitration leads to hepatic injury directly or indirectly through interference of binding between nitrated Hsp90 and CYP2E1, leading to its elevation.

The antioxidant glutathione* S*-transferase 1 (GST-1) was reported to be nitrated at Tyr92 and Tyr152. However, nitration at Tyr92 was shown to be critical in functional change as another example of gain in protein function. This conclusion was evidenced by site directed mutagenesis of each Tyr residue of GST-1, overexpression of each GST-1 mutant in LLC-PK1 cells, and then exposure to peroxynitrite followed by monitoring its activity [41]. The activation of GST-1 nitrated at Tyr92 would improve the antioxidant defense. Tyr nitration was also found to be essential for initiating the seeding process followed by protein aggregation in fibrin for blood clot formation [151] and for aggregation of *α*-synuclein, a component of Lewy bodies in many neurological diseases [54, 55, 152]. In addition, Tyr nitration of the catalytic subunit of protein phosphatase type 2A (PP2A) seems important in reactivating PP2A activity and promoting LPS and interferon-*γ* induced microvascular endothelial barrier dysfunction with increased albumin permeability. This gain of function by nitrated PP2A was resulted from interference with Tyr phosphorylation of the catalytic subunit, which subsequently suppresses PP2A activity [49]. All these examples, especially the antioxidant GST-1 and PP2A, with increased activities and functions through protein nitration, are of interest and one needs to take extra cautions against premature conclusions that protein nitration is considered only as a pathological risk factor without proper evaluation of the enzyme activity and functional consequence.

### 2.3. Induction of Immunogenicity by Nitrated Proteins and Potential Implications in Various Liver Diseases

Another consequence of protein nitration is the induction of immunogenicity and immunomodulation. The body system tolerates its endogenous proteins without eliciting an immune response through immunological tolerance. However, once protein secondary structures are modified via various PTMs, the body immune system might not tolerate the modified proteins and thus immune response might ensue. This immune activation may be due to the structural changes following protein nitration or any other PTMs such as oxidation, carbonylation, phosphorylation, and glycation including AGE, acetylation,* S*-nitrosylation, glutathionylation, and transglutamination. All these PTMs may contribute to the exposure of a new antigenic epitope, leading to stimulation of a cascade of immune response that starts with the activation of B and/or T cells [153]. This immune reaction can be beneficial when under normal conditions where it clears out a harmful complex or can be hazardous if its level becomes abnormal and sustained through a vicious cycle. Thus the aggregated nitrated proteins in various tissues, including the liver, as seen in inflammation, for example, [154], can initiate or exaggerate an inflammatory reaction that might even become chronic, via the stimulation of the immune system against its own proteins, known as an autoimmune response [155]. Indeed, elevated levels of anti-3NT antibodies were reported in the plasma of patients with post-traumatic acute lung injury [156] and in the synovial fluids of patients with rheumatoid arthritis and osteoarthritis as well as in the serum of patients with the autoimmune disease systemic lupus erythematosus [153]. However, Tyr nitration does not always invoke an immune response; instead it might also inhibit the immune response when it seems necessary in some cases. For instance, nitration of T cell receptor—CD8 complex—compromises its ability to bind to the major histocompatibility complex dimers, leading to tolerance of T cells to cancer cells and hence the survival of cancer cells [157, 158]. Thus protein nitration might be involved in immunomodulation, leading to the development of autoimmune diseases and even cancer progression. Since hepatic protein nitration is remarkably elevated in various chronic diseases including inflammatory diseases, aging, and cancers, it would be valuable to carefully evaluate the role of protein nitration in pathophysiology of these diseases. Alternatively, protein nitration can regulate immune function by interfering with Tyr phosphorylation and concurrent activation of various signal transducer and activators of transcription (STAT) proteins, as reported [159, 160].

### 2.4. Lack of Functional Consequences of Nitrated Proteins

Finally protein nitration might not have any significant effect on protein activity, expression, and consequently function, as reported with Tyr nitration of transferrin and *α*1-antichymotrypsin in acute respiratory distress syndrome [161]. It is vital to keep in mind, however, that many studies in the literature with regard to the Tyr nitration or denitration of certain proteins and subsequent functional changes were based on in vitro reactions using high concentrations of peroxynitrite or other nitration-inducing substances and in an environment lacking the full host defense system including various antioxidants present in living organisms. Thus, the nitrative effect of any agent on protein function also needs to be validated in in vivo systems. It is still challenging to determine whether catalytic inhibition or degradation of a particular protein is solely resulted from its Tyr nitration as there are many other forms of PTM that can also inhibit its activity.

Table 1 summarizes some nitrated liver proteins with functional consequences with the reported or suggested consequences.

## 3. Protein Nitration in Mitochondria and Potential Consequences in Various Liver Diseases

Protein Tyr nitration can occur in different cell compartments and play a critical role in hepatic injury. However, based on the aforementioned studies and alterations of key mitochondrial proteins involved in fat and ammonia metabolism, energy supply, antioxidant defense, and so forth (listed in Table 1 and references therein), mitochondrial functional status can become a very important factor in the initiation and/or progression of liver diseases. In addition, NO-dependent inhibition of the mitochondrial respiration was reported in both AFLD and NAFLD [16, 73, 162]. However, most of the reported hepatic studies, with a few exceptions, indicated the pathological role of nitrated proteins in mediating hepatic disease process, relying mainly on the correlation between protein nitration and hepatic injury without in-depth analysis of functional alterations in nitrated mitochondria proteins. Therefore, we briefly describe the formation of protein Tyr nitration and functional consequences that might explain the role of protein nitration in promoting many forms of liver diseases including AFLD and NAFLD.

Under normal physiological conditions, approximately 1%-2% of oxygen leaks out as ROS from the mitochondrial electron transport chain (ETC) [163]. ROS can either combine with protons to produce water [164, 165] or can be handled with mitochondrial antioxidant defense enzymes where these radicals are dismutated by SOD2 and detoxified by glutathione peroxidase (GPX) [165]. Alternatively, these ROS can be involved in various cellular signaling pathways [166]. However, damaged mitochondria in various pathological conditions or exposure to toxic agents such as alcohol, HFD, LPS, abused substances (e.g., MDMA), or other therapeutic drugs (e.g., APAP and AZT) [137, 164, 167, 168], greater amounts of ROS are leaked out from the mitochondrial ETC, possibly at the sites of Complex I and Complex III, as suggested by alcohol-subjected hepatocytes [169]. RNS including NO radicals can also be detected in the mitochondria after NO is produced in cytosol by the NOS isozymes since NO can readily cross the mitochondrial membranes. In addition, mitochondrial RNS can be produced by mitochondria NOS [170]. Thus the excess of ROS and RNS radicals in the mitochondria can lead to the formation of peroxynitrite and hence increased mitochondrial protein Tyr nitration. Mitochondrial protein nitration can also be mediated through the oxidation of Tyr and nitrite via a peroxidase-catalyzed reaction [171, 172]. Mitochondria, which are the major source of cellular ROS, also have low levels of antioxidants such as reduced glutathione (GSH), compared to that in cytosol [173], rendering mitochondria a vulnerable target for nitrative damage. It has also been suggested that rat liver mitochondrial protein turn-over rates were dramatically decreased from days to hours upon exposure to nitrating conditions due to proteolytic degradation [46, 174–176]. Further, mitochondria under nitroxidative stress in animal models or human disease specimens show abnormal and irregular shapes and decreased functions [177, 178]. Nitration of many mitochondrial proteins can further deteriorate mitochondrial functions through inactivation of some critical proteins for cell maintenance and survival. For instance, suppressed activities of antioxidant enzymes such as SOD2, GPX, and GR would lead to decreased antioxidant cell defense, while suppressed ATP synthase may result in decreased ATP production with compromised cellular ability to perform normal functions and increased necrosis. Furthermore, inactivation of thiolase likely leads to the inhibition of the *β*-oxidation of fatty acids with increased hepatic fat accumulation or inefficient supply of an alternative energy (e.g., ketone bodies produced from fat degradation) when glucose supply is decreased during fasting or not efficiently utilized in many disease states. Suppression of mitochondrial aldehyde dehydrogenase 2 (ALDH2), involved in the metabolism of reactive acetaldehyde and 4-hydroxynonenal, would lead to accumulation of reactive aldehydes including lipid peroxidation products ([179], Table 1 and references therein).

Mitochondrial functions can also be compromised via deletion and/or mutation through oxidative/nitrative modifications of mitochondrial DNA, which is important as they encode 13 polypeptides that are all subunits of the 4 mitochondrial ETC proteins (i.e., Complexes I, III, IV, and V) [168, 180]. Mitochondrial DNA is also extremely sensitive to nitroxidative damage due to its location within the cell close to the inner mitochondrial membrane, and the relatively low levels of protective antioxidant enzymes, histone proteins, or polyamines and DNA repair enzymes in mitochondria compared to other subcellular organelles including nuclei ([138, 181] and references therein). In addition, it has been suggested that the rate of mutation in mitochondrial DNA is 10-fold higher than that in the nuclear DNA [182]. Taken together, oxidative damage and/or deletion of mitochondrial DNA [183, 184] may lead to reduced expression and function of mitochondrial ETC proteins, contributing to greater ROS production, as shown in alcohol-exposed rats [70]. Lipid peroxides can further alter the cell membrane functions and promote fibrosis through activation of stellate cells with elevated production of collagen and proinflammatory cytokines/chemokines that promote recruitment of neutrophils and activation of hepatic macrophage Kupffer cells. All these events contribute to profound deleterious effects on mitochondrial functions with increased steatosis, apoptosis, and necrosis. In addition, activation of autophagy and consequent removal of damaged mitochondria by ethanol [185] or APAP [147] have been suggested to be an adaptive and protective pathway against hepatic damage by both substances. The removal of damaged mitochondria under elevated nitroxidative stress may also be a mechanism by which cells can get rid of the nitrated proteins to protect themselves. Collectively, these studies and mechanisms support the notion that oxidative/nitrative stress produces mitochondrial dysfunction that can play a major role in stimulating the damage in the various hepatic diseases including AFLD and NAFLD and also extrahepatic tissue injury including brain diseases [182, 186–188]. Figure 1 summarizes the different pathways that nitrative stress might cause mitochondrial dysfunction, contributing to tissue injury including AFLD and NAFLD.

## 4. Detection Methods of Nitrated Proteins and Challenges

Nitrated proteins should be isolated or enriched before they are subjected to gel electrophoresis separation and protein digestion followed by final MS analysis to identify proteins and possibly Tyr-nitrated peptides. Several methods have been reported to detect Tyr nitration including, but not limited to: (1) two-dimensional gel electrophoresis followed by immunoblot analysis with specific anti-3-NT antibody [189], (2) immunoprecipitation with the specific antibody [47, 190] or immunoaffinity chromatography using immobilized specific anti-3-NT antibody on Sepharose followed by capturing nitrated proteins [191], (3) solution isoelectric focusing, and (4) redox proteomic approach via conversion of nitro-Tyr to amino-Tyr followed by its labeling with biotin or dansyl chloride [192] to isolate nitrated proteins [193, 194]. Recent reviews summarized standard methods for the identification and quantification of proteins nitrated at the Tyr residues through purification of nitrated proteins and in-gel or in-solution trypsin digestion followed by gas chromatography (GC)—or liquid chromatography (LC)—tandem MS analyses [51, 195]. The identification of peptides that originate from nitrated proteins can be performed by using matrix-assisted laser desorption ionization-time-of-flight (MALDI-TOF) MS [51, 195–197], while identification of specific nitrated Tyr residues can be performed by LC-electrospray ionization (ESI)-MS/MS [198]. Despite the fact that all techniques have merits, there are many short outcomes that can be improved especially when it comes to the number of identified proteins and the ability to capture proteins that is not highly expressed or the capturing of nonspecific proteins. Many excellent reviews have been already reported with more details about nitrated protein isolation and characterization protocols and the advantages and disadvantages of each method ([51, 199, 200] and references within).

Our laboratory recently developed a simple approach to efficiently identify and characterize protein nitration and functional consequences. Although the methods of immunoaffinity chromatography were described before [191, 201], the recovery of the nitrated proteins was low. An attempt to improve the method was undertaken by increasing the incubation time of the sample with the immunoaffinity resin to 19 h and by also improving the quality and specificity of captured proteins by using more stringent washing protocol [202]. However, the incubation time in this protocol seems too long and not very practical in purifying sufficient amounts of nitrated proteins for MS analysis especially when nitrated proteins are expressed in very low quantities.

**Figure 2:**
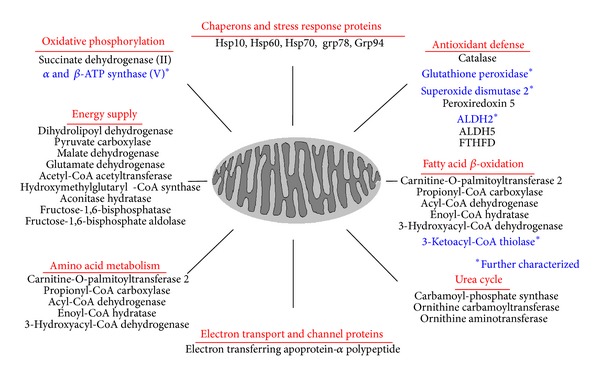
Summary of nitrated mitochondrial proteins in acetaminophen (APAP)-exposed mice. Some recently-identified nitrated mitochondrial proteins in APAP-exposed mouse livers are summarized with respect to the function of each protein identified by mass spectrometry. *Five selected proteins (marked in bold blue) were further characterized for the reversible changes and enzyme activities after APAP exposure in the absence or presence of NAC co-treatment, as described in and adapted from [35].

After preparing tissue homogenates in buffer preequilibrated with nitrogen gas for 1 h to remove dissolved oxygen, the levels of Tyr-nitrated proteins in the target sample compared to control should be determined by immunoblot analysis with anti-3-NT antibody. Once different levels of protein Tyr-nitration between the target and control samples are confirmed, whole homogenates should be further fractionated to prepare lysates from specific subcellular organelles (e.g., mitochondria versus cytosol). Nitrated proteins in mitochondrial extracts from treated and control samples, for instance, are then affinity purified by using the commercially available agarose beads coupled with anti-3-NT antibody, as similar to the method [203]. The affinity-purification steps should be repeated several times to collect sufficient amounts of nitrated proteins. The affinity-purified proteins can be concentrated using mini-spin-column concentrators (Millipore). Small aliquots of purified nitrated samples from control and treated or disease group can be separated on 1D gels and stained with silver to confirm the differences before MS analysis for protein identification. Nitration of some selected proteins identified by MS analysis could be individually confirmed by using immunoprecipitation with the specific antibody against each protein followed by immunoblot with anti-3-NT antibody. In addition, the functional consequence of protein nitration should be determined by measuring the catalytic activity of each target protein [35]. It would be ideal to further determine the nitrated Tyr residue(s) and function by site-directed mutagenesis followed by overexpression and activity measurement. Finally, it is always desirable to determine the causal relationship between Tyr-nitration detected earlier and the full-blown pathological states or tissue injury, usually observed at later time points, following exposure to the toxic substances, as described [35, 140]. Using this method of immunoaffinity purification, we were able to identify more than 30 and 70 nitrated cytosolic and mitochondrial proteins, respectively, in APAP-treated mouse liver. Detection and functional consequences of nitration in five selected mitochondrial proteins (Figure 2 and Table 1) were confirmed via immunoprecipitation followed by immunoblot analysis with anti-3-NT antibody and activity measurements in the absence or presence of an antioxidant, which fully prevented APAP-mediated protein nitration and hepatic damage [35]. Tyr-nitrated peptides of many proteins including SOD2 in APAP-exposed mice could not be identified by the MS analysis. However, nitration of four or five Tyr residues including the critical Tyr34 and inactivation was further confirmed by the MS analysis and activity measurement, respectively, after recombinant SOD2 protein was incubated with a nitrating agent tetranitromethane for 20 min [35].

Based on our recent results, we believe that the immunoaffinity purification followed by MS analysis may represent the best approach among all the aforementioned methods for identifying Tyr nitrated proteins. This approach can be also used to purify sufficient amounts of nitrated proteins for studying the role of protein nitration in various disease models, different organs, and/or different subcellular organelles such as nucleus and endoplasmic reticulum where identification of nitrated proteins can be challenging due to the low yield in isolation of nitrated proteins. However, there are still many challenges with purification and functional characterization of nitrated proteins. Although we and other laboratories reported many nitrated proteins [35, 47, 179, 189–200], and Table 1 it is conceivable that the actual nitrated proteins should be far greater than those we identified in APAP-exposed WT mice. Some nitrated proteins are transiently expressed while the amounts of other nitrated proteins could be decreased possibly through proteolytic degradation upon nitration, as previously mentioned. Therefore, it is difficult to capture and characterize all nitrated proteins analyzed at one time point. By reviewing the literature and based on our own data, we acknowledge that identification of certain nitrated transcription factors, insulin receptors, nuclear receptors, and signaling molecules, for example, could be particularly difficult even after repeated purification steps due to extremely low levels of expression. However, individual characterizations of these factors could be of extreme importance to understand the mechanisms of their well-documented alterations in various disease conditions including AFLD and NAFLD. However, this technical problem could be overcome by immunoprecipitation of the individual protein of interest followed by immunoblot with the specific anti-3-NT antibody and measurement of a functional activity in the absence or presence of a peroxynitrite scavenger. Alternatively, the nitrated proteins of interest could be purified to examine the consequence of their alteration under nitration-inducing conditions. One promising approach could be to employ an in vitro system by using isolated mitochondrial proteins exposed to a nitrating agent or incubated with recombinant NOS. This in vitro method is likely to allow identification of many nitrated mitochondrial proteins. However, the level of peroxynitrite produced by this protocol seems far greater than that generated in in vivo conditions and the in vitro results cannot be used to correlate with samples from animal models or human specimens without extra precaution [46]. Thus there are still many challenges that need to be addressed in future research.

## 5. Translational Research Using Nitration Proteomics Approaches to Evaluate Beneficial Agents to Prevent or Treat Hepatic and Other Tissue Injuries

Upon understanding the potential mechanism(s) of nitroxidative stress-related liver diseases, the next logical extension would be translational application to identify beneficial agents to either prevent or treat the disease progression. The relative levels of nitrated proteins between controls versus disease samples must be evaluated prior to conducting translational research with some protective agents administered before and after or during disease progression in experimental models. We recently reported this kind of translational application by first characterizing the mechanism of APAP-induced tissue injury followed by efficacy evaluation of an agent against liver damage [35, 40, 78]. Firstly, we demonstrated the importance of CYP2E1 and protein nitration in APAP-mediated hepatotoxicity by comparing the phenotypic changes in WT and* CYP2e1*(−/−) mice [40]. In this study, we also showed that one of the mechanisms of nitration-mediated tissue injury was suppression (inactivation) of vital proteins such as SOD1 through ubiquitination and subsequent degradation [35, 40, 78]. These results are consistent with the reports by other scientists [204–207]. In continuation, we then immunoaffinity purified and determined identities of many nitrated proteins in both mitochondria and cytosol at earlier time point (e.g., 2 h after APAP treatment). Five critical enzymes SOD2, GPX, ATP synthase, ALDH2, and thiolase were selected for further characterization with respect to their nitration and activity changes at 2 h post-APAP treatment in the absence or presence of NAC. The results revealed that NAC treatment restored APAP-induced changes in transaminase activities, liver histology, and protein nitration to normal levels. NAC also reversed the inactivation of the five enzymes via Tyr nitration, as evidenced by immunoprecipitation with a specific antibody to each protein followed by immunoblot with anti-3-NT antibody. All these results demonstrate the causal roles of protein nitration in promoting full-blown liver damage usually observed at later time points (e.g., 16 or 24 h after APAP treatment) [35]. The combined results of the two studies [35, 40] about the critical role of protein nitration in APAP-induced liver damage and beneficial effect of NAC are consistent with the reports by other laboratories [208, 209]. Thus, our approaches not only allow the characterization of many nitrated mitochondrial proteins but also provide an opportunity for translational research in evaluating the benefits and mechanisms by which NAC protects from APAP-induced liver injury.

This kind of approach can also be applied to future translational studies on various experimental models or human disease specimens where protein nitration seems to play a key role in promoting disease development while the efficacies of beneficial agents can be evaluated, as exemplified in a model of I-R-related acute liver injury. In this study, the benefit of a peroxynitrite scavenger metalloporphyrin MnTMPyP against I-R-related mitochondrial dysfunction and acute hepatotoxicity was investigated [140]. MnTMPyP pretreatment markedly suppressed the I-R-related elevation of serum transaminase levels, histological damage, iNOS expression, and oxidative modifications of key mitochondrial proteins. MnTMPyP treatment also restored the activities of some essential mitochondria enzymes including ALDH2, thiolase, and ATP synthase inhibited under I-R condition. Based on our recent data [35], the activities of these mitochondrial enzymes in the I-R-mediated acute liver injury model could have been inhibited by protein nitration and then restored in the presence of a peroxynitrite scavenger. For this reason, the levels of nitrated proteins should be also evaluated in the future translational studies when testing the benefit of a protective agent.

Mitochondria are important for energy supply, antioxidant defense, apoptosis, and intermediary metabolism (including ammonia, urea, heme, and so forth) and fatty acid oxidation to provide an alternative energy source of ketone bodies when glucose supply is limited [210–212]. In this regard, it would be of particular importance to characterize the nitrated mitochondrial proteins to gain new mechanistic insights in mitochondrial dysfunction and disease progression in acute and chronic liver diseases in the absence or presence of a protective agent. In addition, the proteomics approach of characterizing nitrated proteins can be very useful in the evaluation of many antioxidants other than NAC such as* S*-adenosylmethionine [213], curcumin [214, 215], and green tea extracts, which decreased nitroxidative stress-mediated development of NAFLD in obese (*ob/ob*) mice [216]. Furthermore, this approach can be used in identifying new disease biomarkers in various organs and various disease stages in experimental models or clinical specimens. For instance, the levels of nitrated proteins in plasma, platelets, and liver tissue were positively correlated with the severity of hepatic cirrhosis [132] and diet-induced obesity [217]. In fact, an excellent review article for identifying potential biomarkers from its discovery to its application in clinical studies has been recently reported [218]. The initial stage is known as the discovery phase with identification of biomarker candidates. Various systems biology approaches including bioinformatics, genomics, proteomics, and metabolomic analysis should be used simultaneously to identify potential biomarkers in experimental animal models or human specimens with a specific disease of interest. The potential biomarkers with relative long half-lives and stabilities have to be evaluated or verified in a large number of patient samples with certain disease states for clinical validation purpose.

## 6. Concluding Remarks

Many reports suggest that PTMs, including Tyr nitration, seem to play an important role, at least partially, in various pathophysiological conditions including AFLD and NAFLD. The causal pathologic role of nitrated proteins, however, was questioned due to their relatively rapid turn-over, degradation, and removal of damaged mitochondria by autophagy. It can be argued that the persistence of the insult-mediated protein nitration over extended periods of time and/or consequent imbalance between protein nitration and turnover can alter protein function. In addition, some of the nitrated proteins may disrupt normal signaling pathways (e.g., insulin signaling pathway), contributing to initiation of a cascade of deleterious events. Alternatively, other nitrated proteins may stimulate hepatic macrophage Kupffer cells with elevated cytokines and chemokines, which promote infiltration of neutrophils and inflammatory tissue injury. When these signaling pathways are initiated, even the removal of nitrative stress or nitrated proteins may not stop the ongoing destructive cascades. These events may explain the fact that certain diseases, albeit depending on disease stages, may not be fully treated or reversed even with the FDA-approved therapeutic drugs.

Nitrated proteins may not only play a role in mediating acute liver injury including DILI but also serve as a biomarker for some hepatic diseases. Due to the many forms of protein PTM, it is still challenging to dissect the unique role of nitrated proteins in promoting certain disease states. Further, many studies relied on positive correlation between protein nitration and injury development without detailed mechanistic studies. Thus, to investigate the role of nitrated proteins, a systematic and comprehensive approach should include identification, biochemical characterization, evaluation of overall consequence, and potential prevention or reversal of nitrated proteins and subsequent tissue injuries in the absence or presence of beneficial agents. This systematic approach would serve as a platform for the development of therapeutic agents against AFLD and NAFLD. However, extra caution should be taken into consideration regarding the functional consequence of any nitrated protein since nitration can also activate certain proteins (e.g., microsomal GST-1), as discussed above. It is also important to use sensitive and advanced methods to cover most peptide areas of a target protein to avoid or minimize the potential overlook in identifying nitrated peptides or proteins.

Protein nitration seems to occur in many subcellular compartments including mitochondria. Because of low levels of glutathione and antioxidant enzymes in mitochondria than in cytosol, mitochondrial lipids, DNA, and proteins can be more susceptible to oxidative and nitrative modifications under increased nitrative stress, leading to mitochondrial dysfunction and tissue injury (Figure 1). In fact, many studies dealt with experimental disease models and clinical specimens from patients showed that mitochondrial proteins are abundantly nitrated and/or oxidatively modified, often accompanied with altered functions. Thus, future studies should lead to better understanding of the underlying mechanism(s) of mitochondrial dysfunction through nitration of many additional mitochondrial proteins and their functional implications in disease development and progression. In addition, the occurrence of protein nitration in different liver cells including hepatocytes, Kupffer cells, endothelial cells, and hepatic stellate cells and their functional implications in various liver diseases are poorly understood although these areas should be characterized further. Furthermore, future translational research should also include the development of mitochondria-directed antioxidants or peroxynitrite scavengers to prevent protein nitration and ultimately treat AFLD, NAFLD, and DILI as well as various diseases in other organs.

## Figures and Tables

**Table 1:** List of confirmed nitrated hepatic proteins and functional consequences.

Nitrated proteins	Activity	Hepatic indication (observed or expected)	References
Carbamoyl phosphate synthase-1 (CPS-1)	Decrease	Hyperammonemia, hepatic encephalopathy	[33]
Glutamine synthetase (GS)	Decrease	Hyperammonemia, hepatic encephalopathy related to sepsis	[34]
3-Ketoacyl-CoA thiolase (Thiolase)	Decrease	Decreased *β*-oxidation of fatty acids with increased hepatic steatosis	[35]
Aldehyde dehydrogenase 2 (ALDH2)	Decrease	Accumulation of acetaldehyde and lipid peroxides with increased aldehyde-related liver toxicity	[35]
Complex I (NADH ubiquinone oxidoreductase)	Decrease	ROS leakage, contributing to decreased energy production and increased apoptosis or necrosis	[36]
Complex V (ATP synthase)	Decrease	Decreased energy production with increased sensitivity toward necrotic liver injury	[11, 35–38]
Cytochrome p450 2E1, B6 (CYP2E1, CYP2B6)	Decrease	Drug metabolism: ROS production and ethanol- and drug-induced liver toxicity	[39]
Cytosolic Cu/Zn-SOD (SOD1)	Decrease	Decreased antioxidant defense with increased drug- or toxin-mediated hepatic damage	[35, 40]
Mitochondrial Mn-SOD (SOD2)	Decrease	Same as above	[35, 41]
Glutathione peroxidase (GPX)	Decrease	Same as above	[35]
Glutathione reductase (GR)	Decrease	Increased oxidative stress with elevated levels of oxidized glutathione	[42, 43]
AKT, IR*β*, IRS-1, IRS-2	Decrease	Decrease insulin signaling with increased hepatic insulin resistance and fatty liver	[44]
CD95	Decrease	Increased hepatic anti-inflammatory defense	[45]
List of nitrated mitochondrial and cytosolic proteins	Not confirmed*	Not confirmed but likely contributing to mitochondrial dysfunction, ER stress, and liver damage	[35]
List of nitrated mitochondrial proteins	Not confirmed	Not confirmed but likely contributing to mitochondrial dysfunction and liver damage	[46]
List of nitrated proteins in different compartments	Not confirmed	Not confirmed but likely contributing to mitochondrial dysfunction, ER stress, and liver damage	[47]
Glutathione-S-transferase (GST)	Increase	Increased hepatic antioxidant defense	[41]
Heat shock protein 90 (Hsp90)**	Increase	Conversion to a toxic protein, contributing to increased liver toxicity	[48]
Protein phosphatase type 2A (PP2A)**	Increase	Increased microvascular endothelial permeability	[49]

*With the exception of the five proteins characterized in detail, as in the reference [35].

**Not confirmed in liver, but expected to occur.

## Abbreviations

3-NT: 3-NitroTyr
AFLD: Alcoholic fatty liver disease
AGE: Advanced glycation end products
ALDH2: Aldehyde dehydrogenase-2
ALS: Amyotrophic lateral sclerosis
CPS-1: Carbamoyl phosphatesynthase-1
CYP2B6: Cytochrome P450 2B6
CYP2E1: Ethanol-inducible cytochrome P450 2E1
DILI: Drug induced liver injury
ER: Endoplasmic reticulum
ETC: Electron transport chain
GR: Glutathione reductase
GS: Glutamine synthetase
GSH: Glutathione
GPX: Glutathione peroxidase
GST-1: Glutathione *S*-transferase-1
HFD: High fat diet
Hsp90: The 90-kDa heat-shock protein
I-R: Ischemia-reperfusion
IR*β*: Insulin receptor-*β*
IRS-1: Insulin receptor substrate
iNOS: Induced nitric oxide synthase
LC: Liquid chromatography
LPS: Lipopolysaccharide
MS: Mass spectrum
NAC: *N*-Acetyl cysteine
NAFLD: Nonalcoholic fatty liver disease
NO: Nitric oxide
PTM: Posttranslational modification
RNS: Reactive nitrogen species
ROS: Reactive oxygen species
PP2A: Protein phosphatase type 2A
PPAR-*α*: Peroxisome proliferator-activated receptor-*α*
SOD1: Cu/ZN superoxide dismutase
SOD2: Mn superoxide dismutase
Thiolase: 3-Ketoacyl-CoA thiolase
Trp: Tryptophan
Tyr: Tyrosine.
